# Socioeconomic Disparities in Disability-Free Life Expectancy and Life Expectancy Among Older Chinese Adults From a 7-Year Prospective Cohort Study

**DOI:** 10.3389/ijph.2022.1604242

**Published:** 2022-07-07

**Authors:** Yuanyuan Zhan, Yaofeng Han, Ya Fang

**Affiliations:** ^1^ State Key Laboratory of Molecular Vaccinology and Molecular Diagnostics, School of Public Health, Xiamen University, Xiamen, China; ^2^ Center for Aging and Health Research, School of Public Health, Xiamen University, Xiamen, China

**Keywords:** older adults, occupation, education, life expectancy, disability-free life expectancy, socioeconomic disparities, economic status

## Abstract

**Objectives:** We examined the magnitude and determinants of socioeconomic disparities in disability-free life expectancy and life expectancy at age 65 (DFLE_65_ and LE_65_) in China.

**Methods:** Data from Chinese Longitudinal Healthy Longevity Survey collected during 2011–2018 (8,184 participants aged ≥65) were used. Socioeconomic status (SES) was measured by economic status (ES), and education, respectively. Multistate Markov models and microsimulations were fitted to estimate DFLE_65_ and LE_65_.

**Results:** LE_65_ between high- and low-ES groups differed by 2.20 years for males and 2.04 years for females. The DFLE_65_ disparity in ES was 1.51 and 1.29 years for males and females, respectively. Not undergoing physical examinations, inadequate fruit/vegetable intake, and stress contributed to 35.10% and 57.36% of DFLE_65_ disparity in ES, as well as 26.36% and 42.65% of LE_65_ disparity for males and females, respectively. These disparities in education and ES were of a similar magnitude, while the above factors contributed little to education disparity.

**Conclusion:** Socioeconomic disparities in DFLE_65_ and LE_65_ existed in China. Physical examination, fruit/vegetable intake and stress partly explained these disparities.

## Introduction

Currently, with a rapidly aging population, more than one billion (approximately 15% of the global population) adults are disabled worldwide [1]. The rate of disability is much higher among older adults [2]. Disability-free life expectancy (DFLE), as a supplement to life expectancy (LE), summarizing disability and mortality experiences, has become an important measure to monitor population health. Many previous studies from high-income countries (HICs) have shown that socioeconomic status (SES), including income, wealth, education and occupation, remains positively associated with LE and DFLE at old ages [3–8]. These socioeconomic inequities in health are inherently unjust and lead to significant financial cost to societies [9, 10]. Reducing such health inequities is a means to improve a whole population’s health and increase healthy aging [11].

As the largest low- and middle-income country (LMIC), China has also confirmed the existence of socioeconomic disparities in DFLE among older adults in 1992–1997 [8]. Since the start of the 21st century, with the accelerated aging, the number of care-dependent Chinese elderly individuals is expected to rise from 25.3 million in 2010 to 66 million in 2050 [12]. Additionally, social security and care systems based on the principles of equitable accessibility and use are undeveloped [13]. This may further aggravate DFLE inequities among older adults. The China-WHO Country Cooperation Strategy 2016–2020 also noted that health inequities will be a key challenge for China in the coming years [14].

However, a recent study from China, using education as an SES indicator, found that the disparity in DFLE at age 65 (DFLE_65_) between literate and illiterate adults was small (0.2 years for males and 0.0 years for females) [15]. Additionally, in contrast to recent studies from HICs [4–6], this study surprisingly found that the proportion of DFLE_65_ in the remaining life among illiterate individuals was higher than that among literate individuals [15]. Other recent studies found an education-mortality gradient among Chinese adults aged 65 years and over [16, 17]. These findings may suggest that there is an education disparity in LE (mortality) but not in DFLE among older Chinese adults. In other words, SES may have a relatively stronger effect on mortality than disability at old ages in China. This study did not estimate a 95% confidence interval (95% CI) for the difference in DFLE and its proportion. Whether this difference is statistically significant when further estimating its 95% CI and whether other SES indicators, such as economic status and occupation, show greater inequality of DFLE or provide additional insights compared to education remain unclear.

Estimating the effect of modifiable risk factors on socioeconomic disparities in DFLE and LE enables setting priorities and implementing policies with realistic targets to reduce these disparities. Many studies have shown that inadequate fruit/vegetable intake, smoking, stress, and inadequate healthcare utilization mainly contributed to the socioeconomic disparity in health [18–22]. However, the health indicators in these studies were mostly focused on LE, mortality, self-reported health, etc. To what extent socioeconomic disparity in DFLE can be reduced by reducing or eliminating these risk factors remains unknown. Do the contributions of these factors to these socioeconomic disparities vary by different measures of SES?

Therefore, we estimated the magnitude and potential determinants of socioeconomic disparities in DFLE and LE among older Chinese adults using different measures of SES.

## Methods

### Study Population

Data from the Chinese Longitudinal Healthy Longevity Survey (CLHLS) [23] were used. The CLHLS is a nationwide population-based longitudinal survey conducted in a sample of randomly selected counties and cities in 23 of the 31 provinces in China. More details about the survey design and data quality are available elsewhere [17, 23]. We used data collected during 2011–2018, which included 3 waves: 2011–2012, 2014, and 2017–2018. The baseline wave (2011–2012) comprised 9,679 participants aged 65 years and over. Individuals without any follow-up after baseline (*n* = 791), with a negative follow-up time (*n* = 180) or a missing disability status (*n* = 499), or without complete data concerning smoking status, fruit/vegetable intake, physical examination, and stress status (*n* = 25) were excluded from analyses (accounting for a total of 15.45%). Thus, the final analytical sample included 8,184 participants. The CLHLS was approved by the research ethics committees of Duke University and Peking University (IRB00001052–13074).

### Socioeconomic Status

Economic status, educational attainment were used to characterize SES. Economic status was measured by the following question: “how do you rate your economic status compared with that of other local people?” and categorized as high (very rich or rich), intermediate (average), or low (poor or very poor). Educational attainment was categorized into low (no schooling), intermediate (1–6 years), and high (7 or more years) by the number of schooling years.

Occupational position was another common indicator being used to assess SES. Occupational position was divided into high (government, institutional and managerial personnel; professional and technical personnel; and military personnel), intermediate (clerks, service industry employees, and manual workers), and low (farmers and the unemployed) according to individuals’ major occupation before age 60. However, about 90% of females in the low occupation and about 81% of males in the low occupation. Occupation may be not much variability in this indicator to pick up exposure to occupational hazards and the like which is what one wants to assess when linking occupation with disability. Therefore, we only displayed the results and discussed the results in discussion, not draw conclusions.

### Disability and Disability-Free Life Expectancy

We divided health state into three states: disability-free, disability, and mortality. Disability was defined as needing assistance in at least one of the activities of daily living (ADL): bathing, dressing, going to the toilet, indoor transferring, continence and feeding. DFLE was defined as expected life years without disability.

### Demographic Characteristics and Risk Factors

Demographic characteristics and impact factors were assessed at every follow-up wave. Individual characteristics included gender (male/female), age, and region (urban/rural). Risk factors included smoking, inadequate fruit/vegetable intake, not undergoing physical examinations, and feeling stress. Smoking status was categorized as smoking and never smoked, with the former category including both currently and formerly smoking. Fruit/vegetable intake was measured with the following question: “Do you eat fresh fruits or vegetables?” It was categorized as daily/almost daily, occasionally, and rarely/no. Inadequate fruit/vegetable intake included “occasionally” and “rarely/no” intake categories. Not undergoing physical examinations was defined as participants not undergoing a regular physical examination once every year. Stress status was assessed with the following question: “Do you often feel fearful or anxious?” It was categorized as feeling stress (always or often) or no stress (sometimes, seldom, or never).

### Statistical Analysis

First, logistic regression models were used to estimate the association of SES with risk factors. A multistate Markov model (MSM) [24] was fitted to estimate the hazard ratios (HRs) for the association of health transitions with SES and risk factors. We fitted 6 MSMs that allowed 4 transitions: from disability-free to disability (disability incidence), from disability-free to death, from disability to disability-free (recovery from disability), and from disability to death. The first model (model 1) adjusted for age, gender, and region. Subsequently, smoking status, fruit/vegetable intake, physical examination status and stress status were entered into model 1 separately as time-dependent covariates (models 2–5) and then simultaneously into model 1 (model 6). In these models, sex-age-region-specific transition probabilities were estimated.

Then, to calculate DFLE_65_and LE_65_, we used microsimulation [25] to simulate a cohort of 100,000 persons at age 65. In this 65-year-old cohort, the distribution of region and gender was sourced from the 2010 census data of China [26], and the gender-region-specific distribution of SES and disability was based on the observed prevalence of the CLHLS’s 2011–2012 wave. From age 65 to death, the health and survival trajectories of each individual in this cohort were governed by the transition probabilities output from the MSM model. The 95% CIs (from the 2.5th and 97.5th percentiles) were estimated by bootstrapping with 1,000 independent replications.

Finally, we set the prevalence of the risk factors to zero in all the SES groups (elimination scenario). We compared the results of the elimination scenario to the results of the current situation to quantify the effect of the risk factors on the socioeconomic disparities in DFLE and LE.

We used economic status and educational attainment as measures of SES to conduct the above analysis separately. These analyses were mainly performed using R software (version 3.5.1) and SAS version 9.4.

## Results

### Characteristics of the Study Population at Baseline

The baseline characteristics of the participants are shown in Table 1. The sample consisted of 3667 males and 4557 females. Of the participants, 19.72%, 26.64%, 28.98% and 24.66% were ages 65–74, 75–84, 84–95, and ≥95 years, respectively. More than half of the participants had low educational attainment (58.82%), only 10.51% had high educational attainment; 15.82% and 17.36% reported low and high economic status, respectively. There were 4349 deaths during 2011–2018. More than a quarter of the older adults were disabled at baseline (26.56%).

**TABLE 1:** Sample characteristics, disability, and death in the Chinese Longitudinal Healthy Longevity Survey, 2011–2018 (China, 2011–2018).

Characteristics	Male (*n* = 3667)	Female (*n* = 4557)	Total (*N* = 8184)
Age group (%)			
65–74	883 (24.08)	731 (16.18)	1614 (19.72)
75–84	1123 (30.62)	1057 (23.40)	2180 (26.64)
85–94	1118 (30.49)	1254 (27.76)	2372 (28.98)
≥95	543 (14.81)	1475 (32.65)	2018 (24.66)
Region (%)			
Urban	1811 (49.39)	2121 (46.96)	3932 (48.04)
Rural	1856 (50.61)	2396 (53.04)	4252 (51.96)
Economic status (%)			
High	686 (18.71)	735 (16.27)	1421 (17.36)
Intermediate	2425 (66.13)	2997 (66.35)	5422 (66.25)
Low	546 (14.89)	749 (16.58)	1295 (15.82)
Missing	10 (0.27)	36 (0.80)	46 (0.56)
Educational attainment (%)			
High	668 (18.22)	192 (4.25)	860 (10.51)
Intermediate	1788 (48.76)	701 (15.52)	2489 (30.41)
Low	1205 (32.86)	3609 (79.90)	4814 (58.82)
Missing	6 (0.16)	15 (0.33)	21 (0.26)
Occupational position (%)			
High	496 (13.53)	120 (2.66)	616 (7.53)
Intermediate	560 (15.27)	319 (7.06)	879 (10.74)
Low	2597 (70.82)	4062 (89.93)	6659 (81.37)
Missing	14 (0.38)	16 (0.35)	30 (0.37)
Disability (%)	750 (20.45)	1424 (31.53)	2174 (26.56)
Death (%)	1878 (51.21)	2471 (54.70)	4349 (53.14)

### Socioeconomic Disparities in DFLE_65_ and LE_65_

Socioeconomic disparities in DFLE_65_ and LE_65_ are displayed in Table 2. A positive socioeconomic gradient was seen in LE_65_ as well as DFLE_65_ when using economic status and educational attainment as measures of SES. LE_65_ between the high and low economic status groups differed by 2.20 (95% CI, 1.10–3.41) years for males and 2.04 (1.01–3.29) years for females. The DFLE_65_ disparity in economic status was 1.51 (0.52–2.59) and 1.29 (0.19–2.52) years for males and females, respectively. Similarly, the LE_65_ disparity between the high- and low-education groups was 2.28 (1.11–3.46) years for males and 1.79 (0.66–3.07) years for females. For DFLE_65_, the education disparity was 1.88 (0.74–2.93) years for males and 1.32 (0.13–2.60) years for females. Overall, the proportion of DFLE_65_ was lower in the high-SES group than in the low-SES group using economic status and educational attainment as markers of SES, although the difference in education was not statistically significant.

**TABLE 2:** Disability-free life expectancy and life expectancy with 95% confidence interval according to socioeconomic status for males and females, based on the Chinese Longitudinal Healthy Longevity Survey, 2011–2018 (China, 2011–2018).

Socioeconomic status	Male			Female		
	DFLE_65_ (years)	LE_65_ (years)	DFLE_65_/LE_65_ (%)	DFLE_65_ (years)	LE_65_ (years)	DFLE_65_/LE_65_ (%)
Economic status						
High	14.44 (13.69, 15.46)	16.63 (15.77, 17.73)	86.83 (85.09, 88.55)	15.48 (14.64, 16.55)	18.71 (17.90, 19.78)	82.74 (80.53, 84.67)
Intermediate	13.54 (13.05, 14.10)	15.27 (14.74, 15.87)	88.67 (87.64, 89.66)	15.00 (14.43, 15.61)	17.68 (17.08, 18.31)	84.84 (83.59, 86.06)
Low	12.93 (12.14, 13.83)	14.43 (13.52, 15.44)	89.60 (87.90, 91.14)	14.19 (13.26, 15.18)	16.67 (15.72, 17.68)	85.12 (83.06, 87.07)
High minus Low	1.51 (0.52, 2.59)	2.20 (1.10, 3.41)	−2.77 (−4.91, −0.55)	1.29 (0.19, 2.52)	2.04 (1.01, 3.29)	−2.39 (−5.16, 0.16)
Educational attainment						
High	14.95 (13.97, 16.01)	16.97 (15.99, 18.07)	88.10 (86.12, 89.71)	15.96 (14.77, 17.24)	19.06 (17.96, 20.38)	83.74 (80.97, 85.83)
Intermediate	13.47 (12.95, 14.12)	15.21 (14.63, 15.92)	88.56 (87.35, 89.64)	15.32 (14.54, 16.11)	18.06 (17.29, 18.89)	84.83 (83.14, 86.33)
Low	13.07 (12.37, 13.84)	14.69 (13.95, 15.48)	88.97 (87.58, 90.28)	14.64 (14.01, 15.27)	17.27 (16.64, 17.93)	84.77 (83.29, 85.89)
High minus Low	1.88 (0.74, 2.93)	2.28 (1.11, 3.46)	−0.87 (−3.00, 1.23)	1.32 (0.13, 2.60)	1.79 (0.66, 3.07)	−1.03 (−3.74, 1.54)
Occupational position						
High	13.49 (12.49, 14.60)	16.17 (15.09, 17.32)	83.43 (80.89, 85.95)	14.08 (12.89, 15.30)	18.05 (16.88, 19.29)	78.01 (74.61, 81.26)
Intermediate	13.35 (12.51, 14.24)	15.59 (14.58, 16.59)	85.63 (83.52, 87.87)	14.04 (13.11, 15.09)	17.74 (16.79, 18.79)	79.14 (76.3, 81.71)
Low	13.73 (13.23, 14.38)	15.26 (14.67, 15.92)	89.97 (89.16, 90.95)	15.01 (14.46, 15.65)	17.57 (16.99, 18.24)	85.43 (84.2, 86.55)
High minus Low	−0.24 (−1.4, 0.78)	0.91 (−0.35, 2.03)	−6.54 (−9.01, −4.21)	−0.93 (−2.14, 0.28)	0.48 (−0.78, 1.60)	−7.42 (−10.65, −4.38)

LE_65_, life expectancy at age 65; DFLE_65_, disability-free life expectancy at age 65; DFLE_65_/LE_65_, proportion DFLE_65_ to LE_65_.

### Socioeconomic Disparities in Prevalence of Risk Factors

**FIGURE 1:**
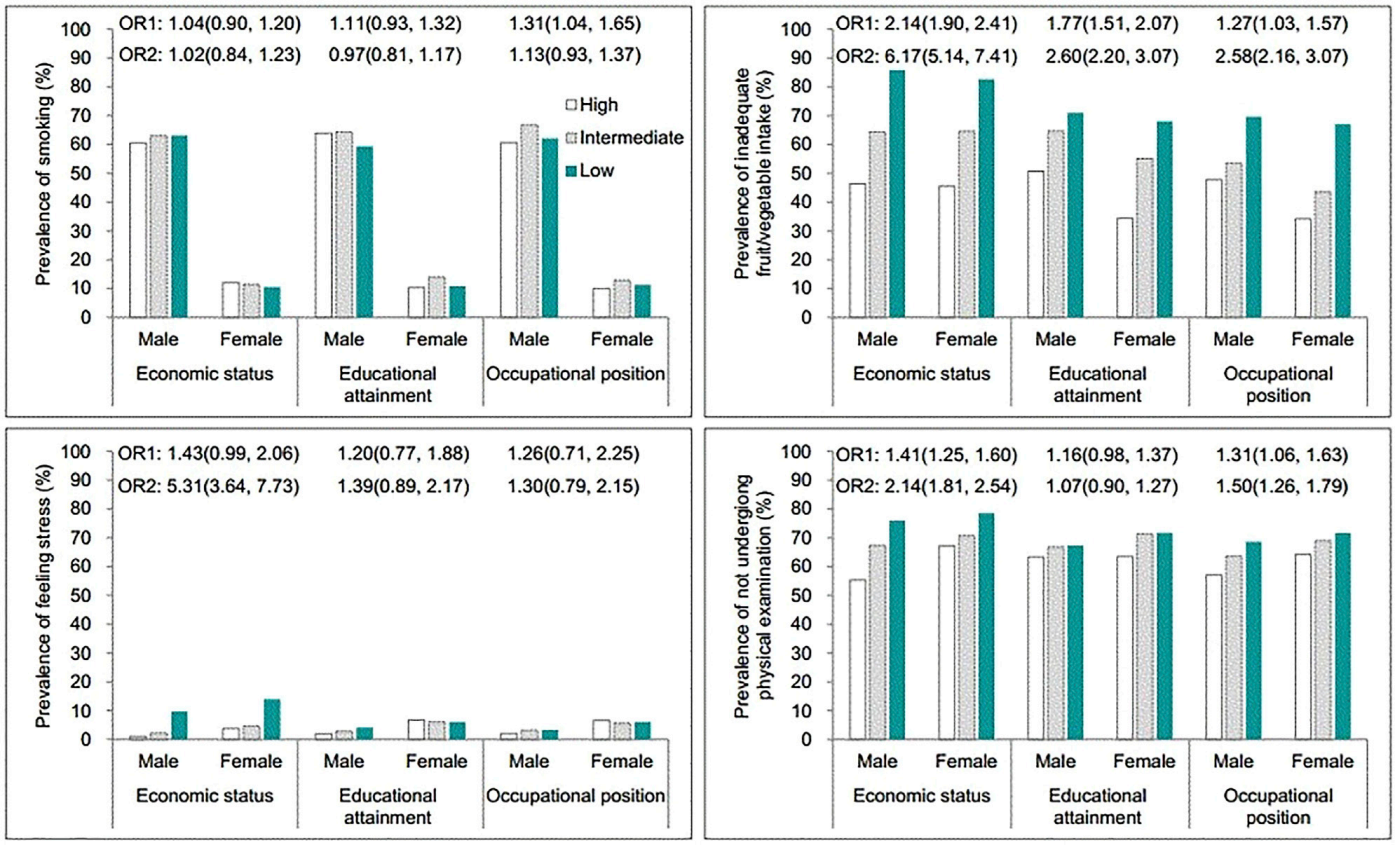
Prevalence of risk factors according to socioeconomic status for older males and females aged 65 years and over, based on the Chinese Longitudinal Healthy Longevity Survey, 2011–2018 (China, 2011–2018).

The prevalence of risk factors according to SES is presented in Figure 1. Economic status and educational attainment were negatively associated with the prevalence of inadequate fruit/vegetable intake (*p* < 0.05), whereas only economic status was negatively related to the prevalence of feeling stress (*p* < 0.001). Lower economic status and educational attainment were associated with higher prevalence of not undergoing physical examinations (*p* < 0.05). Overall, there was little socioeconomic difference in the prevalence of smoking.

### Association of Transitions With Risk Factors

Smoking was the risk factor for disability incidence (HR = 1.20, 95% CI = 1.01–1.43) (Table 3). For mortality from disability-free, not undergoing physical examination was the risk factor (HR = 1.25, 95% CI = 1.05–1.53). For mortality from disability, feeling stress, not undergoing physical examinations, the occasional and rare/no intake of fruit/vegetable were the risk factors, and the HRs (95% CI) were 1.27 (1.08–1.49), 1.11 (1.01–1.22), 1.15 (1.03–1.28), and 1.28 (1.16–1.43), respectively.

**TABLE 3:** The association of transitions with socioeconomic status and risk factors for older adults aged 65 years and over, based on the Chinese Longitudinal Healthy Longevity Survey, 2011–2018 (China, 2011–2018).

Socioeconomic statu/Risk factors	Hazard ratios (95% confidence interval)			
	Disability incidence	Mortality from disability-free	Recovery from disability	Mortality from disability
Panel A				
Economic status (ref = High)				
Intermediate	0.94 (0.79, 1.12)	1.30 (0.97, 1.76)	1.08 (0.77, 1.51)	1.16 (1.03, 1.32)*
Low	0.83 (0.65, 1.05)	1.52 (1.06, 2.17)*	0.95 (0.63, 1.44)	1.28 (1.10, 1.49)*
Educational attainment (ref = High)				
Intermediate	1.07 (0.83, 1.39)	1.33 (0.92, 1.91)	1.02 (0.66, 1.56)	1.06 (0.86, 1.29)
Low	1.35 (1.04, 1.75)*	1.41 (0.97, 2.06)	1.38 (0.89, 2.14)	1.12 (0.92, 1.36)
Occupational position (ref = High)				
Intermediate	0.91 (0.67, 1.24)	1.39 (0.81, 2.37)	1.06 (0.61, 1.83)	0.96 (0.77, 1.20)
Low	0.91 (0.69, 1.16)	1.43 (0.90, 2.29)	1.51 (0.95, 2.38)	1.28 (1.06, 1.54)*
Panel B				
Economic status (ref = High)				
Intermediate	0.93 (0.78, 1.11)	1.27 (0.95, 1.72)	1.07 (0.76, 1.50)	1.13 (1.00, 1.28)*
Low	0.83 (0.65, 1.06)	1.40 (0.97, 2.01)	1.06 (0.69, 1.64)	1.16 (0.99, 1.36)*
Educational attainment (ref = High)				
Intermediate	1.07 (0.83, 1.40)	1.30 (0.91, 1.86)	1.03 (0.67, 1.59)	1.03 (0.84, 1.26)
Low	1.36 (1.04, 1.78)*	1.38 (0.95, 2.00)	1.39 (0.89, 2.17)	1.07 (0.88, 1.31)
Occupational position (ref = High)				
Intermediate	0.90 (0.65, 1.22)	1.37 (0.80, 2.34)	1.06 (0.61, 1.83)	0.94 (0.75, 1.18)
Low	0.88 (0.68, 1.16)	1.36 (0.84, 2.20)	1.47 (0.92, 2.34)	1.23 (1.02, 1.49)*
Panel C				
Smoking status (ref = never smoked)				
Smoking	1.20 (1.01,1.43)*	1.10 (0.88, 1.37)	1.08 (0.8, 1.46)	1.03 (0.92, 1.15)
Fruit and vegetable intake (ref = adequate)				
A little	1.00 (0.85, 1.18)	1.05 (0.83,1.34)	1.03 (0.78,1.36)	1.15 (1.03, 1.28)*
Little	0.92 (0.78, 1.09)	1.2 (0.94, 1.52)	0.88 (0.65, 1.18)	1.28 (1.16, 1.43)*
Stress status (ref = no stress)				
Feeling stress	1.23 (0.90, 1.69)	0.86 (0.44, 1.67)	0.60 (0.33, 1.06)	1.27 (1.08, 1.49)*
Physical examination status (ref = undergoing)				
Not undergoing	1.00 (0.87, 1.15)	1.25 (1.03, 1.53)*	0.78 (0.61, 0.99)	1.11 (1.01, 1.22)*

Socioeconomic status was measured by economic status, educational attainment, and occupational position. Panel A: Hazard ratios were obtained from multistate Markov models adjusted for age, gender, and region. Panel B: Hazard ratios were obtained from multistate Markov models adjusted for age, gender, region, smoking, stress, fruit/vegetable intake, and physical examination. Panel C: Hazard ratios were obtained from the multistate Markov model mutually adjusted for age, gender, region, smoking, stress, fruit/vegetable intake, and physical examination.^*^ *p* < 0.05.

### Association of Transitions With SES

The association of transitions with SES and the risk factors are shown in Table 3. For mortality from disability-free, the HR (95% CI) for low economic status (high as reference) was 1.52 (1.06–2.17) in the model adjusted for age, gender, and region. After adjusting smoking, fruit/vegetable intake, physical examination, and stress, the HR was attenuated to 1.40 (0.97–2.01). Similarly, for mortality from disability, after adjusting these factors, the HR for low economic status decreased from 1.28 (1.10–1.49) to 1.16 (0.99–1.36), and the HR for intermediate economic status slightly decreased from 1.16 (1.03–1.32) to 1.13 (1.00–1.28). The association of economic status with disability incidence and recovery had no statistical significance (*p* > 0.05).

Low education was only associated with disability incidence (HR = 1.36, 95% CI = 1.04–1.78). After adjusting for the above factors, the HRs changed little.

### Contribution of Risk Factors to Socioeconomic Disparities in DFLE_65_ and LE_65_

**FIGURE 2:**
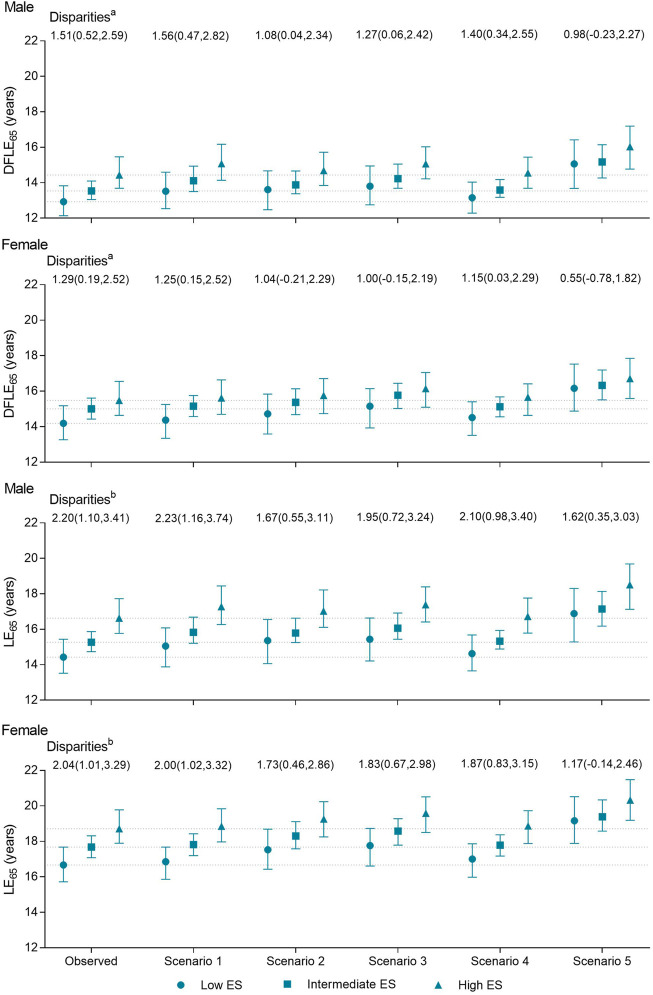
Observed and scenario socioeconomic disparities in disability-free life expectancy and life expectancy, based on the Chinese Longitudinal Healthy Longevity Survey, 2011–2018 (China, 2011–2018).

The DFLE_65_ and LE_65_ disparities in economic status were substantially reduced by eliminating not undergoing physical examinations or inadequate fruit/vegetable intake and somewhat reduced by eliminating feeling stress, but these disparities changed little by eliminating smoking (Figure 2). For example, the DFLE_65_ disparity between males with high and low economic status was reduced from 1.51 (0.52–2.59) years to 1.08 (0.04–2.34) years by eliminating inadequate fruit/vegetable intake. That is, 28.48% of the DFLE_65_ disparity in economic status for males was attributed to inadequate fruit/vegetable intake. Inadequate fruit/vegetable intake, not undergoing physical examinations, and stress contributed in total to 35.10% of the DFLE_65_ disparity in economic status for males and 57.36% for females. The contribution from these factors to the LE_65_ disparity in economic status was 26.36% and 42.65% for males and females, respectively. However, the contribution of these risk factors to education disparities in DFLE_65_ and LE_65_ was small (approximately 10%) (Table 4).

**TABLE 4:** Socioeconomic disparities in disability-free life expectancy and life expectancy with 95% confidence interval, based on the Chinese Longitudinal Healthy Longevity Survey, 2011–2018 (China, 2011–2018).

Socioeconomic status/Gender	DFLE_65_ disparity (high minus low)			LE_65_ disparity (high minus low)		
	Observed	Scenario	Contribution	Observed	Scenario	Contribution
Male						
Economic status	1.51 (0.52, 2.59)	0.98 (−0.23, 2.27)	35.10%	2.20 (1.10, 3.41)	1.62 (0.35, 3.03)	26.36%
Educational attainment	1.88 (0.74, 2.93)	1.71 (0.49, 3.02)	9.04%	2.28 (1.11, 3.46)	2.02 (0.72, 3.48)	11.40%
Occupational position	−0.24 (−1.40, 0.78)	—	—	0.91 (−0.35, 2.03)	—	—
Female						
Economic status	1.29 (0.19, 2.52)	0.55 (-0.78, 1.82)	57.36%	2.04 (1.01, 3.29)	1.17 (-0.14, 2.46)	42.65%
Educational attainment	1.32 (0.13, 2.60)	1.19 (-0.16, 2.58)	9.85%	1.79 (0.66, 3.07)	1.57 (0.25, 3.00)	12.29%
Occupational position	−0.93 (−2.14, 0.28)	—	—	0.48 (−0.78, 1.60)	—	—

Socioeconomic status was measured by economic status, educational attainment, and occupational position. Contribution was calculated as (Observed-Scenario)/Observed×100%.Scenario assumes that no older adults have any of the risk factors (smoking, inadequate fruit/vegetable intake, not undergoing physical examinations and feeling stress).LE_65_, life expectancy at age 65; DFLE_65_, disability-free life expectancy at age 65.

## Discussion

The results yield 4 main findings. First, socioeconomic disparity in DFLE_65_ and LE_65_ existed in China in 2011–2018. The magnitudes of these disparities were similar when using economic status and education as markers of SES. Second, the socioeconomic disparity in LE_65_ was larger than that in DFLE_65_, and the higher-SES group had a lower proportion of DFLE_65_ to LE_65_. Third, economic status was negatively associated with mortality at old ages, but educational attainment was negatively related to disability incidence. Fourth, inadequate fruit/vegetable intake, not undergoing physical examinations and stress played important mediating roles in the DFLE_65_ and LE_65_ disparities in economic status but not in this disparity in education.

In contrast to another Chinese study (3-year follow-up) that found little DFLE_65_ disparity in education [15], our results showed a greater education-DFLE_65_ gradient. This inconsistency may be because our study had a longer follow-up time (7 years). Studies on socioeconomic disparities in DFLE and LE at old ages have been reported in other countries. In Denmark, the DFLE_65_ disparities between tertiary education and primary/lower secondary education were 2.9 and 3.4 years for males and females in 2014, respectively; for LE_65_, the disparities were 2.4 and 2.2 years [6]. The income disparities (highest and lowest income quintiles) in DFLE_65_ (6.1 years for males and 5.6 years for females) and LE_65_ (4.7 years for males and 3.3 years for females) seemed larger than the education disparities [4]. In England and the United States, the socioeconomic disparities in DFLE were larger for wealth than for education and occupation [3]. The disparities between the richest (in the top 33% of wealth) and poorest (in the bottom 33% of wealth) were 7–8 years at age 60 and 6–7 years at age 70 in 2002–2013 [3]. In 10 western European countries, the average education difference (lower secondary education or lower versus tertiary education) in DFLE_65_ was 4.6 years for males and 4.4 years for females; the average LE_65_ disparity was 3.0 years for males and 1.9 years for females in 1995–2001 [5]. In Japan, the education difference (≤9 years of formal education versus >9 years of formal education) in DFLE_65_ was 2.4–2.5 years, and in LE_65_ it was 2.1 years for people who were active at baseline in 1999–2009 [27]. Although our results cannot be directly compared with the results from these studies because of different measures of SES and disability, the magnitudes of DFLE_65_ and LE_65_ disparities in economic status and education in China appeared similar to those in Japan but smaller than those in western developed countries.

Unlike economic status and education, occupational position seemed to be negatively associated with DFLE_65_, although the association was not statistically significant. Furthermore, higher occupational position was related to higher disability prevalence (Supplementary Figure S1). There are two plausible explanations for these puzzling findings. First, most of the older adults with low occupational positions were farmers and still needed to do farm work to survive at older ages, which may contribute to better physical functioning. A study revealed that farm work was negatively related to dependency duration in late life [28]. Second, older adults with low occupational positions had higher mortality from disability. The mortality selection made older adults with low occupational positions pass away at early ages, and the people who survived to old ages had healthier physical functioning [29]. Occupational position was categorized into 3 groups (high, intermediate, low) with about 90% of females in the low occupation and about 81% of males in the low occupation, which may lead to an underestimation of the effect.

Consistent with another recent study from China [15], the proportion of DFLE_65_ was lower in the higher-education group. However, it lost statistical significance when we further estimated a 95% CI. However, we found that the proportion of DFLE_65_ was lower in the higher-SES group when using economic status as markers of SES and that the disparity in LE_65_ was larger than that in DFLE_65_. Moreover, economic status were associated with mortality but not with disability incidence and recovery (Table 3). These results suggested that SES had a stronger effect on mortality than disability at old ages in China. The lower mortality in the lower-SES group merits further study.

Additionally, economic status was negatively associated with disability prevalence at age 65 (Supplementary Figure S1). These results indicated that the effect of economic status on disability may mainly occur in early life rather than in late life. A Netherlands study showed that the average age at onset of disability in a low-SES group (62 years for males and 61 years for females) was younger than that in a high-SES group (76 years for males and 75 years for females) [30]. Implementing relevant policies targeting early-life disability and late-life mortality among those with low SES may greatly improve DFLE and reduce health inequities in later life.

Unlike in some countries where smoking has a great effect on health disparities [31–33], smoking had little effect on DFLE_65_ and LE_65_ disparities in SES in China. Consistent with previous studies, stress was an important mediator of the association between economic status and health [21, 34]. However, the effect of stress on the DFLE_65_ and LE_65_ disparities in economic status was small for its relatively lower prevalence among older adults. It was inadequate fruit/vegetable intake and not undergoing physical examinations that were the determinants of these disparities in economic status and should be given priority when making policies to reduce the inequities in DFLE and LE.

However, inadequate fruit/vegetable intake, not undergoing physical examinations and stress had little effect on the educational disparities. Unlike economic status, educational attainment seemed to affect DFLE_65_ and LE_65_ through different paths. Educational attainment was negatively associated with disability incidence but not mortality at old ages. A previous study of 7 LIMCs showed that approximately two-thirds of disabilities were attributed to chronic disease [35]. Another study of 20 HICs and LIMCs revealed that all-cause mortality and cardiovascular events were negatively associated with education but not with health [36]. These findings may suggest that educational attainment may affect DFLE_65_ and LE_65_ through chronic disease. In addition, the older adults with higher education may get a better job or better know the rules of cardiovascular prevention, such as reducing the consumption of salt or cigarette smoke or body weight, to have better health outcomes.

### Strengths and Limitations

Our study provided updated results on the socioeconomic disparities in DFLE and LE among older adults using different measures of SES in the 2010s in an LMIC. Moreover, the SES and risk factors may consequently change with time. Therefore, we used repeated measures of SES and risk factors as time-dependent variables to estimate the effect of risk factors on this disparity. A study has suggested that the effect of risk factors will be underestimated if only risk factors are assessed at the first follow-up [33].

However, there are some limitations. First, risk factors were self-reported and categorized broadly on two or three levels, which potentially underestimated their effects. Second, we did not use income or wealth to assess SES due to its potential multiple sources (e.g., cumulative income throughout life, monetary assistance from family members and various sideline economic activities) and the difficulty of obtaining accurate accounts. Second, economic status was self-perceived and subjective. However, subjective SES, such as self-perceived economic status and adequacy of income, has also been used to assess the association of SES with health in previous studies [37–39]. Subjective SES has been thought to capture differences in wealth [40]. Furthermore, self-perceived economic status may have been more related to relative SES within the respondents’ region. Some of the high-SES people from rural areas, for instance, may have had lower absolute SES than intermediate-SES people from the cities. Local governments may find it effective to make scientific decisions targeted to relatively poor older adults within their regions. Third, losses to follow-up and from nonresponse may have biased our results. The excluded participants tended to live in rural areas and have lower economic status than the eligible participants, but the difference was small (Supplementary Table S1). Fourth, confounding factors that were unknown or not included in the analysis, such as moderate or heavy drinking, may have contributed to an overestimation of outcomes. However, it was difficult to obtain alcohol consumption in our data. Finally, the MSM model we fitted based on the Markov process assumed that the transitions between health statuses depended only on the present status, not on the sequence of events that preceded it and thus did not account for individual heterogeneity in disability status history.

In conclusion, DFLE_65_ and LE_65_ disparities existed in economic status and education. Moreover, the LE_65_ disparity was greater than the DFLE_65_ disparity. The mechanisms of the DFLE_65_ and LE_65_ disparities in economic status and education differed. Physical examinations, fruit/vegetable intake and stress had great effects on DFLE_65_ and LE_65_ disparities in economic status but not in education. Future policies should pay more attention to older adults’ economic status and education and use different interventions for different groups.

## Supplementary Material

Supplementary Figure S1 Disability prevalence across socioeconomic status (China, 2011–2018).
