# Supplementary material for: Socioeconomic Disparities in Disability-Free Life Expectancy and Life Expectancy Among Older Chinese Adults From a 7-Year Prospective Cohort Study
Source: Int J Public Health. 2022 Jul 7;67:1604242. doi: 10.3389/ijph.2022.1604242 (PMC9302194; doi:10.3389/ijph.2022.1604242)
Supplement: Supplementary file 1 [file Table1.DOCX]

| **Table S1** Difference between eligible and excluded samples | | | | | |
| --- | --- | --- | --- | --- | --- |
| Characteristics | | Eligible sample (*n*=8184) | Excluded sample (*n*=1495) | *OR* for being excluded | *P* |
| Demographics | Gender |  |  |  |  |
|  | Male | 3667(44.81) | 665(44.48) | ref |  |
|  | Female | 4517(55.19) | 830(55.52) | 1.085 | 0.284 |
|  | Region |  |  |  |  |
|  | Urban | 3932(48.04) | 679(45.42) | ref |  |
|  | Rural | 4252(51.96) | 816(54.58) | 1.294 | <0.001 |
|  | Age group |  |  |  |  |
|  | 65-74 | 1614(19.72) | 313(20.94) | ref |  |
|  | 75-84 | 2180(26.64) | 367(24.55) | 0.896 | 0.205 |
|  | 85-94 | 2372(28.98) | 402(26.89) | 0.899 | 0.224 |
|  | >=95 | 2018(24.66) | 413(27.63) | 1.049 | 0.602 |
| SES | Economic status |  |  |  |  |
|  | High | 1421(17.46) | 218(15.20) | ref |  |
|  | Intermediate | 5422(66.63) | 1013(70.64) | 1.333 | <0.001 |
|  | Low | 1295(15.91) | 203(14.16) | 1.248 | 0.049 |
|  | Missing | 46 | 61 |  |  |
|  | Educational attainment |  |  |  |  |
|  | High | 860(10.54) | 207(13.97) | ref |  |
|  | Intermediate | 2489(30.49) | 453(30.57) | 0.872 | 0.193 |
|  | Low | 4814(58.97) | 822(55.47) | 0.804 | 0.056 |
|  | Missing | 21 | 13 |  |  |
|  | Occupational position |  |  |  |  |
|  | High | 616 (7.55) | 152(10.24) | ref |  |
|  | Intermediate | 879(10.78) | 240(16.16) | 1.129 | 0.340 |
|  | Low | 6659(81.67) | 1093(73.6) | 0.620 | <0.001 |
|  | Missing | 30 | 10 |  |  |
| Risk factors | Smoking | 2790(34.44) | 447(32.32) | 0.870 | 0.058 |
|  | Inadequate fruit/vegetable intake | 5209(64.30) | 848(61.32) | 0.930 | 0.252 |
|  | Feeling stress | 382 (4.72) | 50 (3.62) | 0.991 | 0.891 |
|  | Not undergoing physical examination | 5602(69.15) | 951(68.76) | 0.767 | 0.089 |
| OR was obtained from logistic regression models. OR, odds ratio. | | | | | |
