# Supplementary figures and images for: Socioeconomic Disparities in Disability-Free Life Expectancy and Life Expectancy Among Older Chinese Adults From a 7-Year Prospective Cohort Study
Source: Int J Public Health. 2022 Jul 7;67:1604242. doi: 10.3389/ijph.2022.1604242 (PMC9302194; doi:10.3389/ijph.2022.1604242)

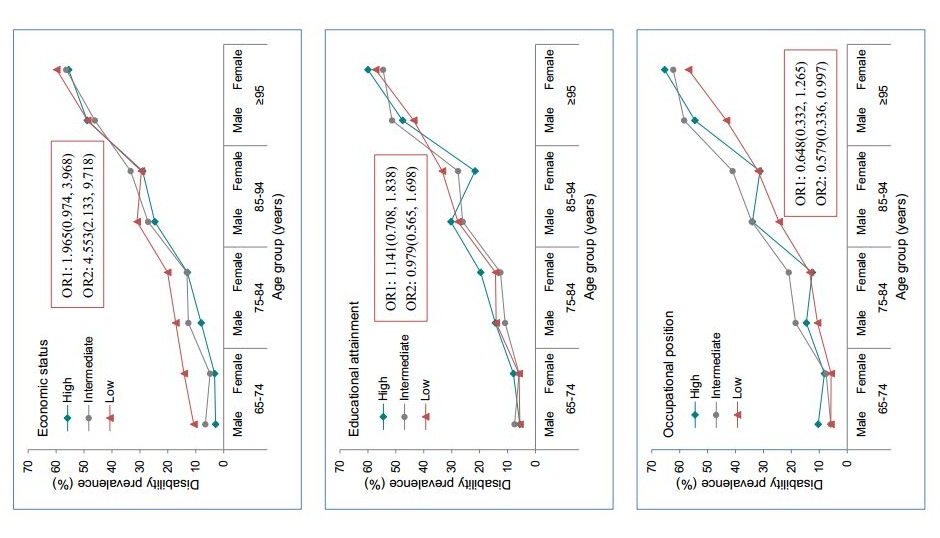

Supplement: Supplementary file 2 [file Image1.JPEG]
